# Effects of Four Sulfonate-Containing Additives and Hydroxyethyl Cellulose on the Properties of Electrolytic Copper Foils

**DOI:** 10.3390/molecules30020229

**Published:** 2025-01-08

**Authors:** Wei Wang, Jun Tao, Kaiwen Tong, Zhiqiang Xu, Fuqi Zhong, Jianping Dong, Yanxia Chen, Zhengbing Fu, Caiqin Qin

**Affiliations:** 1 Jiangxi XinboRui Technology Co., Yingtan 335000, China; weiwang@hbeu.edu.cn (W.W.); tj@xbrkj.com (J.T.); tjwen2025@163.com (K.T.); 15273268006@163.com (F.Z.); jpdong0128@163.com (J.D.); yanxiachen2024@163.com (Y.C.); 2 School of Chemistry and Materials Science, Hubei Engineering University, Xiaogan 432000, China; 3 Hubei Key Laboratory of Biological Resources and Environmental Biotechnology, Wuhan University, Wuhan 430000, China

**Keywords:** sulfonated organics, hydroxyethyl cellulose, electrolytic copper foil, tensile strength, pinhole

## Abstract

Ultrathin electrolytic copper foils with a thickness of 6 μm were prepared by a test machine using copper sulfate electrolyte with gelatin, hydroxyethyl cellulose (HEC), and sulfonic acid-containing organics as additives. The effects of four sulfonic acid-containing organic additives, sodium 3-mercaptopropanesulfonate (MPS), bis-(sodium sulfopropyl)-disulfide (SPS), sodium 3-[[(dimethylamino)thioxomethyl]thio]propanesulfonate (DPS), and sodium 3-((4,5-dihydrothiazol-2-yl)thio)propane-1-sulfonate (TPS), on the physical property of copper foils were investigated. The results show that all these additives can effectively improve the gloss and tensile strength of electrolytic copper foil, and the texture coefficients of Cu(111) selectivity increase. The synergistic use of HEC and TPS can effectively reduce the pinholes of copper foil.

## 1. Introduction

Lithium-ion batteries have the advantages of high energy density, long cycle life, and no pollution, and are one of the most widely used secondary energy sources in commercialization [1,2,3]. Electrolytic copper foil is used as a negative current collector in lithium-ion batteries, carrying the active substance and conducting current [4,5,6,7,8]. The tensile strength, elongation, and densification of copper foil have an important influence on the negative electrode production process and the electrochemical performance of batteries. Lithium batteries, especially power lithium batteries, have high energy density requirements. In the case of other conditions remain unchanged, the thinner and lighter the lithium copper foil, the lighter the mass of the lithium battery, and the more the amount of active material contained in the unit mass of the battery, so that the lithium-ion battery energy density has been improved [9]. The thinner the copper foil and the more coating, the more force the copper foil is subjected to during the coating process, and naturally, the higher the requirements for its physical properties, especially tensile strength and elongation.

Optimizing the preparation process of copper foil, including adjusting basic electrolysis parameters and modifying additive formulations, is the optimal choice for regulating the physical properties of copper foil and ensuring the quality of copper foil products. Kondo et al. [10] investigated the effect of current density on the nucleation process of copper electrodeposition and showed that at a current density of 10 mA/cm^2^, the copper foils grew in a pyramidal shape parallel to the substrate and formed optimally oriented the Cu(200) texture of the copper foil. At a current density of 50 mA/cm^2^, the Cu foil grows in the shape of a slanting platelet on the substrate, forming the Cu (111) texture of the copper foil with the best orientation. The effect of temperature and stirring on copper nucleation was investigated by Dutra et al. [11]. It was shown that the initial stages of copper nucleation and growth are closely related to potential and temperature. Still, the effect of stirring will cause copper nucleation only at very high potentials. Lin et al. [12] investigated the effect of flow rate on the surface morphology of copper, and the results showed that under the action of strong convection, the strong convection can weaken the uneven distribution of MPS caused by the current distribution, thus contributing to the surface flattening of electrolytic copper foil. Additives can change the microstructure and morphology of the plated layer without affecting the conductivity of the plating solution. Most of the additives are through cathodic adsorption [13,14,15], complexation with metal ions [16], affect the diffusion of metal ions [17,18], and other effects to hinder or promote the nucleation and growth process of the grains in the cathodic interface, and thus regulate the brightness, tensile strength, and elongation and other properties of copper foil. The effects of pinholes in copper foils on batteries are manifold, including degradation of battery performance, reduction in safety, shortening of cycle life, and reduction in reliability [19,20,21]. Hydroxyethyl cellulose is a macromolecular polysaccharide composed of glucose, which can improve the wettability of the electroplating solution. G. Yi et al. [19] have demonstrated that HEC can effectively inhibit the formation of pinholes. Sulfonate-containing organics are generally used as brighteners [12,22,23,24,25,26]. Sulfur-containing organics selectively adsorb onto specific crystal faces on the surface of the deposited layer, hindering unidirectional crystal growth and inhibiting cone or block formation of copper. Synergistic use with other additives, such as gelatin or hydroxyethyl cellulose, can refine the copper foil crystals to achieve smooth and bright results. Therefore, it is necessary to control the quality of the copper foil and the production process by selecting suitable additives.

**Figure 1:**
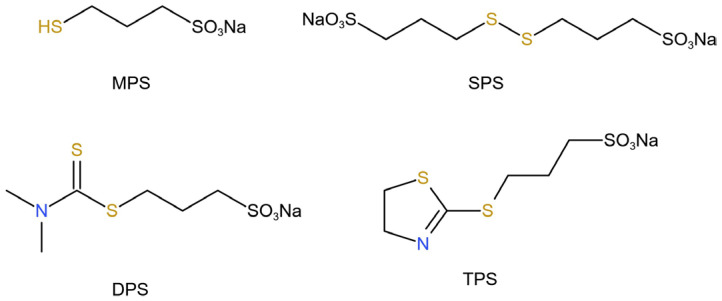
Schematic molecular structures of four sulfonate-containing organics.

In this paper, electrolytic copper foils with a thickness of 6 μm were obtained by electrodeposition on a test machine using an acidic copper sulfate electrolyte with sulfonate and gelatin as additives. The effects of four sulfonic acid-containing organics (Figure 1), Sodium 3-mercaptopropanesulfonate (MPS), Bis-(sodium sulfopropyl)-disulfide (SPS), Sodium 3-[[(dimethylamino)thioxomethyl]thio]propanesulfonate (DPS), and sodium 3-((4,5-dihydrothiazol-2-yl)thio)propane-1-sulfonate (TPS) on the surface morphology and mechanical properties of copper foils were investigated, as well as the effect of hydroxyethyl cellulose on the reduction in pinholes.

## 2. Results

### 2.1. Effect of Four Additives on Gloss and Roughness of Electrolytic Copper Foils

The following ratios were used as the basic electrolyte to obtain 6 um copper foils: Cu^2+^ (90–95 g/L), H_2_SO_4_ (100–105 g/L), Cl^−^ (20–25 mg/L), Gelatin (15 mg/L). Glossiness not only enhances the visual aesthetics of copper foil but also reflects the fineness of its production process. Table 1 shows the effect of adding four additives at different concentrations on the gloss and roughness of copper foils. The gloss of copper foil obtained without sulfate addition is 19 GU, and the roughness is 2.599 μm. It can be found that the gloss of copper foil increases with the increase in additives. MPS has the greatest effect on the glossiness of copper foil, which increases to 159 GU at 0.6 mg/L and 291 GU at 3.0 mg/L. Similar to MPS, increasing the concentration of the other sulfonate-containing additives (SPS, TPS, and DPS) can effectively improve the gloss of copper foils. A total of 263 GU of gloss was obtained when SPS was added at a dosage of 3.0 mg/L, and 243 GU and 215GU of gloss were obtained when the dosage of 4 mg/L was added to DPS and TPS, respectively. The roughness of copper foil is also one of the important surface properties of copper foil, which can effectively increase its electrical conductivity, solderability, corrosion resistance, and adhesion properties with other materials. The test results showed that the addition of four kinds of sulfur-containing additives can effectively reduce the roughness of copper foil. The roughness of copper foil can be reduced to 1.597 μm with the addition of 3 mg/L of MPS and 1.506 μm with the addition of 1 mg/L of SPS, and the roughness of copper foil can be reduced to even lower with the addition of DPS and TPS. The roughness of the copper foil was only 1.211 μm with the addition of 5 mg/L DPS and 1.449 μm with the addition of 4 mg/L TPS, which may be related to the N element in DPS and TPS.

As Table 1 shows, all four sulfur-containing compound brighteners can regulate the brightness and roughness of the copper foil surface. The probable reason is that the brightening agents can selectively adsorb copper on specific crystal surfaces and inhibit the growth of copper grains [13,14,15]. Together with the action of other additives, such as gelatin [27], the copper foil can be ade to achieve the effect of brightness and low roughness.

### 2.2. Effect of Four Additives on Tensile Strength and Elongation at Break of Electrolytic Copper Foils

**Figure 2:**
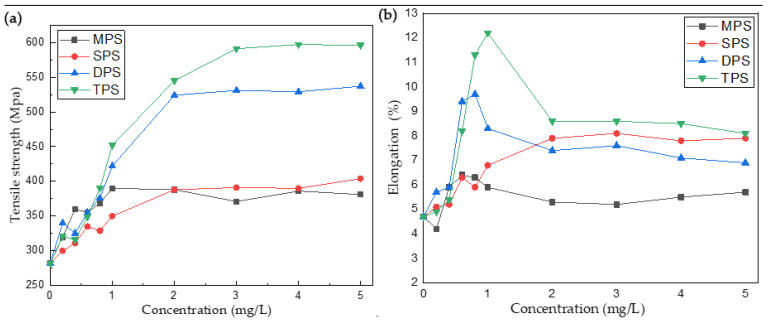
(**a**) Effect of four additives on tensile strength of copper foil; (**b**) effect of four additives on elongation at break.

Batteries are subjected to thousands of charging and discharging experiments, which puts higher requirements on the tensile strength and elongation at the break of copper foil. Table 2 lists the effects of four additives on the tensile strength and elongation at the break of copper foil at different concentrations. As Figure 2a shows, the tensile strength of copper foil without sulfide as an additive is 282 MPa, and the elongation at break is 4.7%. Copper foil’s tensile strength reaches 380 MPa when MPS is added at 2.0 mg/L, but its tensile property decreases slightly with the increase in concentration. The tensile strength of the copper foil reaches 404 MPa when MPS is added at 5.0 mg/L. Compared with MPS, the tensile strength of the copper foil can be further improved by adding DPS and TPS. The tensile strength of the copper foil can reach 531 MPa when the addition of DPS is 3 mg/L, and the tensile strength of the copper foil can reach 597 MPa when the addition of TPS is 4 mg/L. The experimental results show that the addition of these four sulfur-containing additives can improve the tensile strength of copper foil. As can be seen from Figure 2b, these four sulfur-containing additives also enhance the elongation of copper foil. The addition of MPS can increase the elongation of copper foil slightly. When MPS is added at a concentration of 0.6 mg/L, the tensile strength of copper foil is 355 MPa, and the elongation is up to 6.4%, but the elongation decreases when the concentration continues to increase. When the concentration of SPS reaches 3.0 mg/L, the tensile strength of copper foil is 391 MPa, and the elongation is 8.1%. The elongation is unchanged when the concentration of SPS continues to increase. When DPS was added at a concentration of 0.8 mg/L, the tensile strength of the copper foil was 376 MPa, and the elongation was 9.7%. Similar to DPS, the tensile strength of copper foil was 390 MPa, and elongation was 11.3% at a concentration of 0.8 mg/L of TPS. At a concentration of 1.0 mg/L of TPS, the tensile strength of the copper foil was 390 MPa, and the elongation increased to 12.2%. Continuing to increase the concentration of TPS, the tensile strength of copper foil continued to increase, but the elongation decreased.

The tensile strength of the copper foils prepared with MPS and SPS is about 400 MPa, while the higher tensile strength and elongation of the copper foils prepared with DPS and TPS may be related to the structure of the four additives. The chemical structures of the four additives contain sulfonate, SPS contains a -SH group, MPS contains a -S-S- group, DPS contains a -R_2_N-C=S and a -S- group, and TPS contains a five-membered heterocyclic ring and a -S- group. The N element in DPS and TPS can form ammonium ions, which can be adsorbed on the active sites (bumps) on the surface of the electrodes [28], and the adsorption layer has a hindering effect on the precipitation of copper. The adsorption layer has a hindering impact on the precipitation of copper, causing copper to be deposited there. The adsorption layer hinders the precipitation of copper, so copper deposition is inhibited here. The deposition of copper is inhibited here, while the deposition of copper elsewhere is normal. This reduces the peak heights and raises the valleys on the surface of the copper foil, which becomes flat and improves the physical properties.

### 2.3. Effect of Four Additives on Surface Morphology and Crystal Texture Orientation

**Figure 3:**
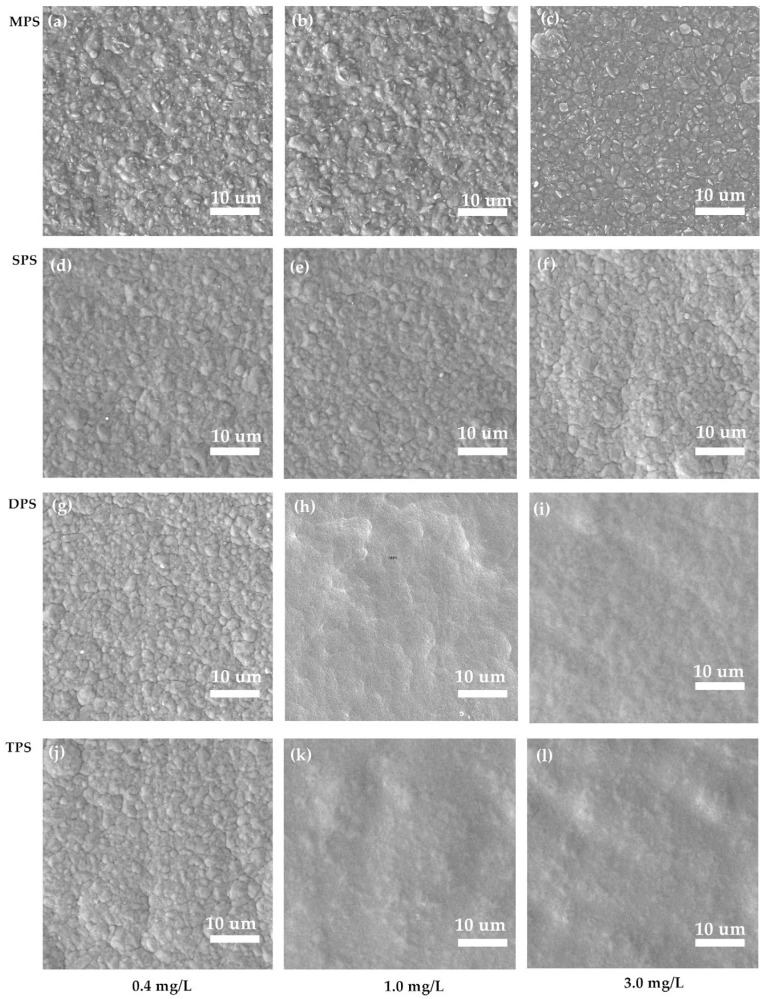
SEM images of copper foils with four additives at different concentrations. (**a**) 0.4 mg/L of MPS, (**b**) 1.0 mg/L of MPS, (**c**) 3.0 mg of MPS, (**d**) 0.4 mg/L of SPS, (**e**) 1.0 mg/L of SPS, (**f**) 3.0 mg of SPS, (**g**) 0.4 mg/L of DPS, (**h**) 1.0 mg/L of DPS, (**i**) 3.0 mg of DPS, (**j**) 0.4 mg/L of TPS, (**k**) 1.0 mg/L of TPS, (**l**) 3.0 mg of TPS.

**Figure 4:**
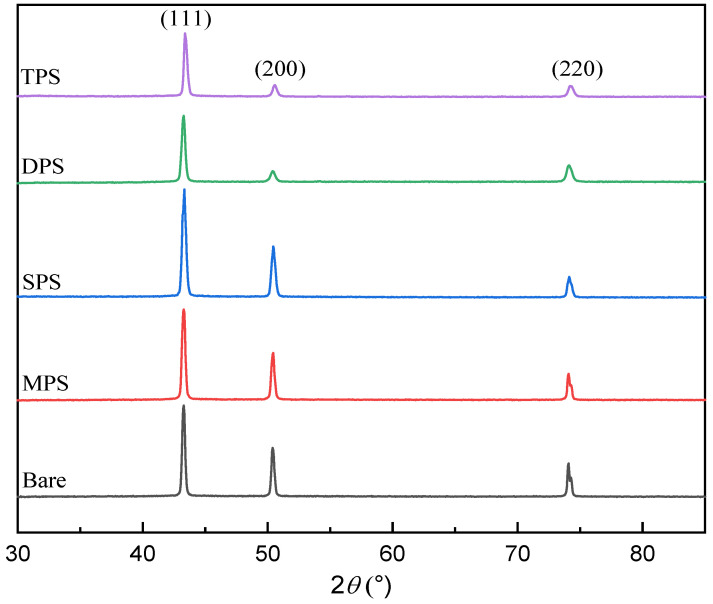
XRD analysis of copper foil prepared by the four additives.

The scanning electron microscope (SEM) images of the gross surfaces of the copper foils prepared by the four additives at different concentration conditions are shown in Figure 3. From the figure, it can be observed that a large number of particles existed on the surface of the copper foil when the concentration of the additives was at a low level, and the size distribution of these particles showed a large variability. When using the MPS additive, even when its concentration was increased by 3.0 mg/L, the size difference between particles was still more obvious. The effect of adding SPS is similar to that of MPS; the surface of copper foil becomes flat, but the size distribution of copper particles is still not uniform. When adding DPS and TPS additives, the effect is similar to SPS. With the gradual increase in the concentration of additives, the gross surface of the copper foil becomes relatively flat, and the particles on the surface of the copper foil gradually tend to be uniformly distributed. SEM analyses show that adding these four additives can refine the grains, and the effect of DPS and TPS refining the grains is better. To study the effect of the structure of four kinds of sulfur-containing organics on the mechanical properties of copper foils, the samples with the highest tensile strength of each of the four additives were selected and analyzed by X-ray diffraction, and the XRD of copper foils obtained from the four additives are shown in Figure 4. Table 3 describes the texture coefficients of the crystalline surfaces of copper foils obtained by adding different additives. The characteristic diffraction peaks of the copper foil appear at 42.8°, 49.9°, and 74.0°, which correspond to the (111), (200), and (220) crystal planes of face-centered cubic copper, respectively. The values of TC(111), TC(200), and TC(220) for the copper foils obtained without the addition of sulfonate-containing compounds were 35.00%, 27.16%, and 37.83%, respectively. After the addition of MPS, the values of TC(111) and TC(200) of Cu foil were slightly increased. The TC(111) of the copper foil reaches 43.3% with an increase of 8.83% with the addition of SPS compared to bare, and the value of TC(200) also increases slightly, while the value of TC(220) decreases to 27.03%. With the addition of the DPS additive, the crystalline surface of Cu(111) shows a dominant orientation, and the value of TC(111) increases to 47.00%. In comparison, the value of TC(200) decreases to 14.78%. After the addition of TPS, the diffraction intensity of the Cu(111) crystal plane was further increased, and the value of TC(111) was increased to 51.38%, while the texture coefficients of the other crystal planes were decreased. Compared with the additives without sulfonate-containing additives, the values of TC(111) were significantly increased with the addition of all four additives, in the range of 37.7%–51.5%, showing the Cu(111) crystalline facets selectively oriented. Combined with the molecular structures of the additives, it is speculated that functional groups such as -N=C and C=S can promote the growth of (111) crystalline surface of copper foil.

### 2.4. Effect of HEC on Copper Foil Pinholes

**Figure 5:**
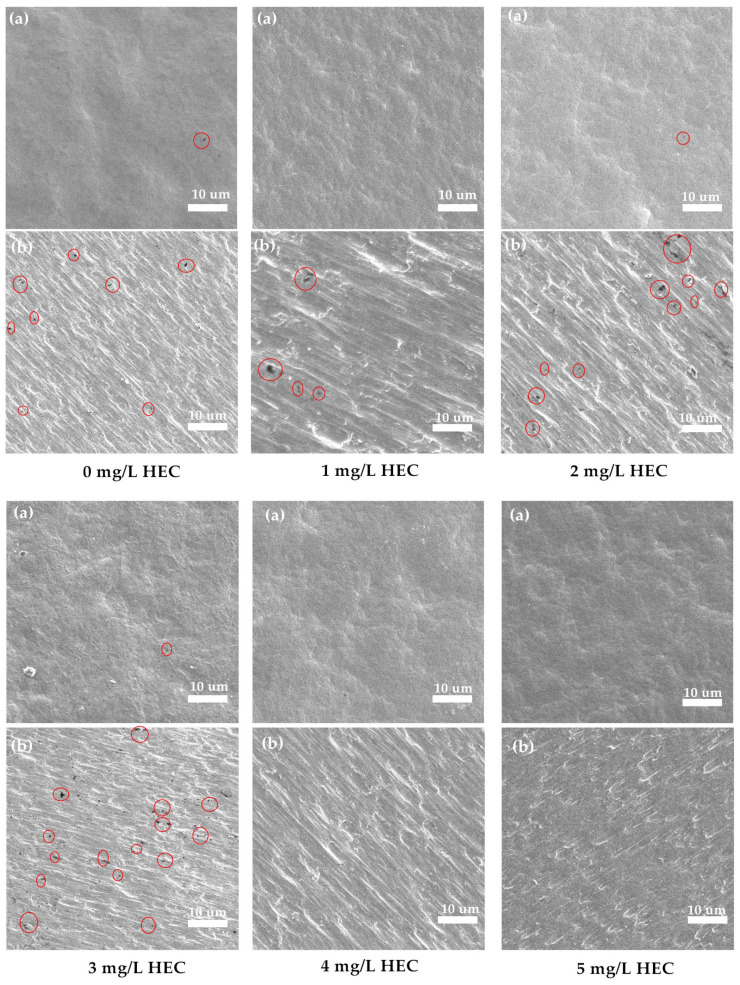
SEM analysis of copper foil surfaces with different concentrations of HEC added (Red circles indicate pinholes). (**a**) Rough surface, (**b**) glossy surface.

The occurrence of pinholes can seriously affect the performance and effectiveness of copper foils. Pinholes in copper foils are usually closely related to the fineness of the manufacturing process, the quality of the raw materials, the accuracy of the production equipment, and the control of the production environment. Among them, adjusting the electrolyte formulation is regarded as an effective strategy to solve the copper foil pinhole problem. In this study, we have tried to use carboxyethyl cellulose (HEC), which is used in conjunction with TPS, to reduce pinholes in copper foil. The formulation for producing 6 μm copper foil is as follows: Cu^2+^ (90–95 g/L), H_2_SO_4_ (100–105 g/L), Cl^−^ (20–25 mg/L), Gelatin (15 mg/L), TPS (3.0 mg/L), HEC (1.0–5.0 mg/L). As shown in Figure 5, a small number of pinholes existed on the surface of the prepared copper foil when HEC was not added to the electrolyte. With the increase in HEC concentration, the number of pinholes on the surface of the copper foil showed an increase and then a decrease until it disappeared. When the concentration of HEC reaches 3.0 mg/L, the number of pinholes is the highest; when the concentration of HEC increases to 4.0 mg/L, no pinholes can be seen on the surface of the copper foil, and when it increases to 5.0 mg/L, the pinhole phenomenon disappears. The experimental results show that the moderate addition of HEC can effectively reduce the generation of pinholes on the surface of copper foil.

Next, we tested the physical properties of copper foils prepared with additives of different HEC concentrations. As can be seen from the data in Table 4, Figure S1 and Figure S2, compared with the copper foils prepared without HEC, the copper foils prepared with HEC showed a decrease in surface gloss and roughness, and the tensile strength also tended to weaken. In contrast, the elongation showed a trend of decreasing and then increasing. The copper foil obtained by adding 5.0 mg/LHEC had a brightness of 142 GU, a gross surface roughness of 1.155 μm, a tensile strength of 564 MPa, and an elongation of 6.1%.

**Figure 6:**
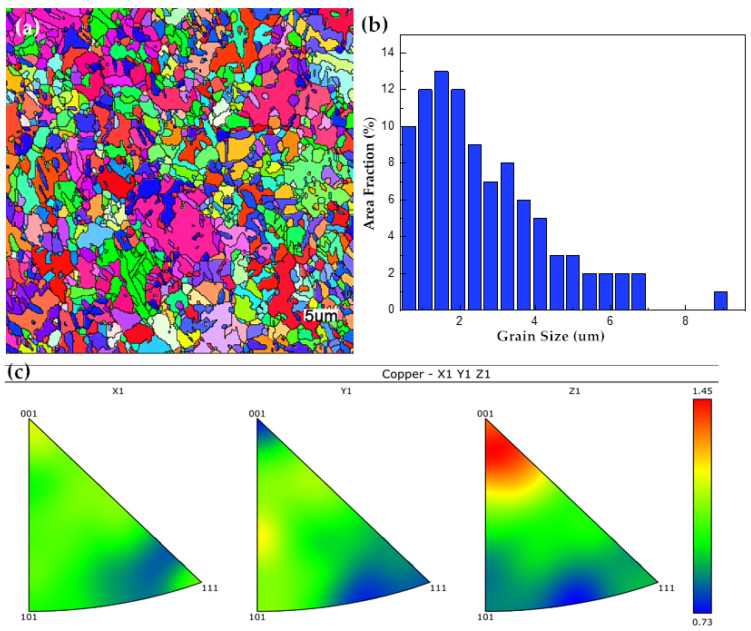
The EBSD test results of copper foils prepared by adding 5 mg/L of HEC. (**a**) IPF map, (**b**) grain diagram, (**c**) reverse polarity map.

Table 5 describes the preferential texture coefficients of the crystalline surfaces of copper foils prepared by adding different concentrations of HEC additives. We observed that the texture coefficient of Cu(111) gradually decreases with increasing concentration of HEC additions, while the proportion of Cu(200) texture coefficient gradually increases. After adding 5.0 mg/L of HEC, the values of TC(111), TC(200), and TC(220) of Cu foil were 32.00%, 35.26%, and 32.74%, respectively, and the surface of the copper foil was at the same level in different directions. The XRD analysis of copper foils prepared by adding different concentrations of HEC is shown in Figure S3. Figure 6 and Figure S4 show the EBSD test results of copper foils prepared by adding 5.0 mg/L of HEC. It can be observed that most of the grain diameters are below 7 µm with an average of 1.5 µm. Slightly different from the XRD test results, the dominant crystal surface orientation of the copper foil is Cu(200) crystal surface.

## 3. Discussion

**Figure 7:**
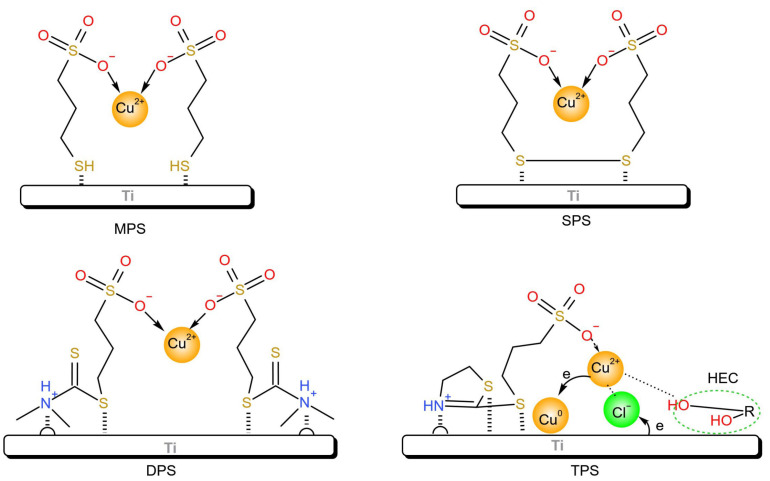
Possible mechanisms of action of adsorption by four additives.

As shown in Figure 7, the sulfhydryl group at one end of the MPS molecule is used for adsorption on the surface of the cathode roller to form an adsorption layer [29]. When increasing the concentration of MPS, the growth of the nuclei of the copper adsorbed by MPS receives a hindrance, thus favoring the refinement of the copper grains. Continuing to increase the concentration of MPS leads to an excess of copper ions near the cathode roll, the rapid growth of copper grains, the copper grains become larger, and the strength of the prepared copper foils to pull the rope decreases instead. Excessive amounts of the additive MPS lead to difficulties in desorption and migration as well as uneven distribution, resulting in localized overgrowth and increased roughness of the copper foil grains. The mechanism of action of SPS is similar to that of MPS, and the two additives may be converted to each other during the electrolysis process [30]. Similar to MPS, SPS possesses two sulfur molecules that can be adsorbed on the surface of the cathode rolls by electronic forces to increase the coverage area. With an increase in SPS concentration, more locations will be covered, and the tensile strength of the prepared copper foils will be a little greater than that of MPS. DPS has a thiourea structure and can act as a leveling agent [31]. It forms ammonium cation in the acidic electrolyte, can be attached to the bumps of cathode rolls, and is easy to accept electrons near the cathode so that the tensile strength of the prepared copper foil can be further improved. However, the thiourea group may make the copper foil brittle during the preparation of copper foil, so it limits the use of DPS [32]. The TPS structure contains a thiazole ring. Both ammonium ions and S atoms can be adsorbed with the cathode roll. The thiazole ring can be adsorbed on the copper surface to increase the surface coverage, which is conducive to the inhibition of copper deposition. Refined grains are easy to obtain, thus improving the tensile strength of the prepared copper foils [33].

Hydroxyethyl cellulose is a macromolecular polysaccharide composed of glucose, which can improve the wettability of the electroplating solution. Adding HEC can increase the viscosity of the electrolyte, improve the wetting performance of the plating solution, reduce the uneven deposition of copper foil caused by poor electrolyte flow, and thus reduce the probability of pinhole formation. When the concentration of HEC is 1.0–3.0 mg/L, the film formed on the surface of the cathode roll is not uniform due to the low concentration, which blocks the precipitation of copper ions and leads to the increase in pinholes; when the concentration of HEC is 4 mg/L, the HEC can be adsorbed to the surface of the cathode roll more uniformly, and the directional arrangement occurs on the surface of the cathode to make the grains uniform, which effectively reduces the formation of pinholes on copper foil. At the same time, HEC can reduce the surface tension at the interface of the cathode roll, promote the discharge of gas generated by a hydrogen precipitation reaction, and avoid generating pinhole defects. HEC can form a “-Cu^2+^-Cl^−^” adsorption structure, and TPS can seize copper ions and then precipitate copper in the vicinity of the TPS so that the precipitated copper crystals are more detailed and the tensile strength is higher. The experimental results show that HEC and TPS together can effectively reduce the pinholes in copper foils, increase the texture coefficients of Cu(200) crystal surface texture, and maintain the elongation at about 7%, which is consistent with the experimental results of B. Fan [34].

## 4. Materials and Methods

### 4.1. Materials

2-Mercaptothiazoline (AR, 98%), 1,3-Propanesultone (AR, 99%), Gelatin (from bovine skin, gel strength ~300 g Bloom), NaOH(AR, 99%), CH_3_CH_2_OH (AR, 99.5%), H_2_SO_4_ (AR, 98%), HCl (AR, 37%), CuSO_4_·5H_2_O (AR, 99%), sodium 3-mercaptopropanesulfonate (AR, 99%), bis-(sodium sulfopropyl)-disulfide (AR, 99%), sodium 3-[[(dimethylamino)thioxomethyl]thio]propanesulfonate (AR, 99%). All of the above reagents were purchased from Energy Chemical. Copper standard solutions (NCS Testing Technology Co., Ltd., Beijing, China).

### 4.2. Preparation of Sodium 3-((4,5-dihydrothiazol-2-yl)thio)propane-1-sulfonate (TPS)

**Figure 8:**
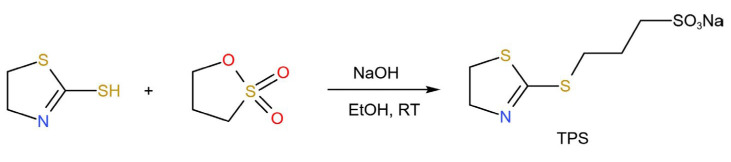
Reaction equation for the preparation of TPS.

The reaction equation for the preparation of TPS is shown in Figure 8. In a 50 mL flask, 1.19 g (0.01 mol) of 2-mercaptothiazoline and 0.48 g (0.012 mol) of NaOH were added to 10 mL of ethanol and completely dissolved, followed by the addition of an ethanol solution containing 1.22 g (0.01 mol) of 1,3-propanesulfonolactone dropwise over 20 min. The reaction was continued for 4 h. The reaction mixture was filtered to remove the solvent, and the resulting solid mixture was recrystallized to give the product TPS. The structure of the product TPS was verified by NMR in 86% yield. Figure S5 shows the NMR spectrum of sodium 3-((4,5-dihydrothiazol-2-yl)thio)propane-1-sulfonate (TPS). The FTIR spectrum of sodium 3-((4,5-dihydrothiazol-2-yl)thio)propane-1-sulfonate (TPS) and 2-mercaptothiazoline are shown in Figures S6 and S7, respectively.

TPS: ^1^H NMR (400 MHz, D_2_O, ppm) *δ* = 4.35–4.31 (t, *J* = 8.64 Hz, 2H), 3.81–3.77 (t, *J* = 8.64 Hz, 2H), 3.47–3.44 (t, *J* = 7.38 Hz, 2H), 3.04–3.00 (m, 2H), 2.26–2.19 (m, 2H).

^13^C NMR (100 MHz, D_2_O, ppm) *δ* = 194.29, 54.12, 48.91, 33.89, 32.91, 24.00.

### 4.3. The Basic Components of Electrolytes

The experiments were based on fine-tuning this formulation.

Cu^2+^ (90–95 g/L), H_2_SO_4_ (100–105 g/L), Cl^−^ (20–25 mg/L), Gelatin (15 mg/L).

### 4.4. Electrodeposition Process for the Preparation of Copper Foils

Copper foils were prepared by electrodeposition using a titanium roller as cathode (3.00 m × 1.58 m) and a half-arc iridium-plated titanium anode with the following process parameters: plating solution flow rate of 50–55 m^3^/h, temperature of 50–55 °C, current density of 32,000–40,000 A/m^2^. The copper layer was deposited on the surface of the cathode roll by controlling the cathode current density during the electrodeposition process, the cathode roll was rotated continuously, and the copper foil was peeled off and then wrapped around the winder roll after passing through the guide roll, and the thickness of the copper foil produced was about 6 μm.

### 4.5. Analytical Methods

Scanning electron microscopy (SEM, JEOL, JSM-6510, Tokyo, Japan) was used to test the morphology of the copper foil microspheres. Electron backscatter diffraction (EBSD, NordlysMax3, Oxford, UK). X-ray photoelectron spectroscopy (XPS, ESCALAB 250 xi, Thermo, Waltham, MA, USA) was used to Analyze the crystalline orientation of copper foil gross surfaces. Intelligent Gloss Meter (GM, SMN 268, Qingdao Topco, Qingdao, China) was used to measure the gloss of copper foils. A Roughness Meter (RM, M300C, MarSurf, Göttingen, Germany) was used to test the roughness of copper foils. An electronic universal testing machine (EUT, AG-IS/1KN, Shimadzu, Kyoto, Japan) was used to test the tensile strength and elongation at the break of copper foil. The structure of TPS was examined by Nuclear Magnetic Resonance (Bruker Avance III 400 Hz, Berlin, Germany).

### 4.6. Performance Test

A square mold of (100 ± 0.2) mm was used to take samples from the left, middle, and right parts of the copper foil in the direction of width and mark them well, and a surface roughness meter, an analytical balance, and a gloss meter were used to test the surface roughness (the roughness of glossy and roughness of hairy surfaces were recorded as Ra and Rz, respectively), the quality and gloss, and the average values were taken.

Samples were taken in the direction of the width of the lower roll, cut to length (152 ± 0.5) mm and width (13 ± 0.25) mm, and tested for elongation and tensile strength on a tensile testing machine with a chuck displacement rate of 50 mm/min.

The texture of the copper foils was analyzed by X-ray diffractometer, and the texture coefficient of different crystalline planes was calculated according to Equation (1).

(1) $$ΤC_{\left(hkl\right)}=\frac{I_{\left(hkl\right)}/I_{0(hkl)}}{\sum \left(I_{\left(hkl\right)}/I_{0(hkl)}\right)}\times 100\%$$

where *h*, *k*, *l* represent the diffraction of the crystal plane index; TC*_(hkl)_* represents (*hkl*) crystal plane of the optimal orientation coefficient; *I*_0(*hkl*)_ is the standard copper powder (*hkl*) crystal plane of the diffraction intensity; and *I*_(*hkl*)_ is the copper foil (*hkl*) crystal plane of the diffraction intensity.

## 5. Conclusions

Adding additives sulfonic-containing to the electrolyte can refine the grain size of copper foil, improve the tensile properties of copper foil, and improve the surface gloss. The combined use of TPS and HEC can effectively reduce the pinholes in the copper foil, and when the concentration of the TPS is 3.0 mg/L and the concentration of the HEC is 4.0 mg/L, the tensile strength of the 6 um copper foil prepared is 578 MPa, and there are no pinholes. As a next step, we will continue to develop copper foils with better physical properties by adjusting the formula of additives.

## Figures and Tables

**Table 1:** Gloss and roughness at different additives.

Entry		MPS	SPS	DPS	TPS
	mg/L	Gloss (GU)/Roughness (Rz, μm)	Gloss (GU)/Roughness (Rz, μm)	Gloss (GU)/Roughness (Rz, μm)	Gloss (GU)/Roughness (Rz, μm)
1	0	19/2.599	19/2.599	19/2.599	19/2.599
2	0.2	56/2.941	40/2.439	32/2.521	35/2.670
3	0.4	103/2.538	78/2.628	71/2.611	63/2.456
4	0.6	159/2.438	121/2.210	100/1.930	86/2.643
5	0.8	191/2.338	162/1.981	110/1.770	96/2.005
6	1.0	222/1.899	234/1.506	130/1.821	112/1.974
7	2.0	258/1.756	238/1.511	158/1.762	123/1.643
8	3.0	291/1.597	263/1.745	220/1.609	181/1.743
9	4.0	287/1.701	266/1.587	243/1.302	215/1.449
10	5.0	285/1.688	265/1.698	236/1.211	216/1.586

**Table 2:** Tensile strength and elongation at break with different additive concentrations.

Entry		MPS	SPS	DPS	TPS
	mg/L	Tensile Strength (Mpa)/Elongation at Break (%)	Tensile Strength (Mpa)/Elongation at Break (%)	Tensile Strength (Mpa)/Elongation at Break (%)	Tensile Strength (Mpa)/Elongation at Break (%)
1	0	282/4.7	282/4.7	282/4.7	282/4.7
2	0.2	320/4.2	300/5.1	340/5.7	321/4.9
3	0.4	360/5.9	311/5.2	325/5.9	316/5.4
4	0.6	355/6.4	335/6.3	355/9.4	349/8.2
5	0.8	369/6.3	329/5.9	376/9.7	390/11.3
6	1.0	370/5.9	350/6.8	422/8.3	452/12.2
7	2.0	388/5.3	388/7.9	524/7.4	545/9.6
8	3.0	371/5.2	391/8.1	531/7.6	591/8.6
9	4.0	366/5.5	390/7.8	529/7.1	597/8.5
10	5.0	361/5.7	404/7.9	537/6.9	596/8.1

**Table 3:** The texture coefficients (TC) of copper foils prepared with four additives.

Entry	TC(111)/%	TC(200)/%	TC(220)/%
Bare	35.00	27.16	37.83
MPS (2.0 mg/L)	37.78	28.51	33.71
SPS (3.0 mg/L)	43.32	29.65	27.03
DPS (5.0 mg/L)	47.00	14.78	38.22
TPS (4.0 mg/L)	51.48	17.19	31.33

**Table 4:** Physical properties of copper foils prepared at different concentrations of HEC.

Entry	Gloss (GU)	Roughness (Rz, μm)	Tensile Strength (Mpa)	Elongation at Break (%)
HEC (1.0 mg/L)	262	1.743	571	7.6
HEC (2.0 mg/L)	212	1.654	567	5.4
HEC (3.0 mg/L)	196	2.023	554	4.7
HEC (4.0 mg/L)	152	2.345	578	7.4
HEC (5.0 mg/L)	142	1.155	564	7.1

**Table 5:** The texture coefficients of copper foils prepared with four additives.

Entry	TC(111)/%	TC(200)/%	TC(220)/%
HEC (1.0 mg/L)	53.31	19.35	27.33
HEC (2.0 mg/L)	46.22	22.75	31.03
HEC (3.0 mg/L)	43.54	31.10	25.35
HEC (4.0 mg/L)	42.96	32.03	25.01
HEC (5.0 mg/L)	32.00	35.26	32.74

## Data Availability

Supporting data can be obtained from the corresponding authors.

## Supplementary Materials

The following supporting information can be downloaded at https://www.mdpi.com/article/10.3390/molecules30020229/s1. Figure S1: Effect of HEC on brightness and roughness of copper foils; Figure S2: Effect of HEC on tensile strength and elongation at break of copper foils; Figure S3: XRD analysis of copper foils prepared by adding different concentrations of HEC; Figure S4: The polarity map of copper foils prepared by adding 5.0 mg/L of HEC. Figure S5. The NMR spectrum of sodium 3-((4,5-dihydrothiazol-2-yl)thio)propane-1-sulfonate (TPS); Figure S6. The FTIR spectrum of sodium 3-((4,5-dihydrothiazol-2-yl)thio)propane-1-sulfonate (TPS); Figure S7: The FTIR spectrum of 2-mercaptothiazoline.
