# Supplementary material for: Effects of Four Sulfonate-Containing Additives and Hydroxyethyl Cellulose on the Properties of Electrolytic Copper Foils
Source: Molecules. 2025 Jan 8;30(2):229. doi: 10.3390/molecules30020229 (PMC11768062; doi:10.3390/molecules30020229)
Supplement: Supplementary file 1 [file molecules-30-00229-s001.zip › molecules-3381203-supplementary.pdf]

Supplementary information

Effects of Four Sulfonate-Containing Additives and Hydroxyethyl Cellulose on the Properties of Electrolytic Copper Foils

Wei Wang [1,2,3], Jun Tao [1], Kaiwen Tong [1], Zhiqiang Xu [1], Fuqi Zhong [1], Jianping Dong [1], Yanxia Chen [1], Zhengbing Fu [1,2,*] and Caiqin Qin [2,3,*]

[1] Jiangxi XinboRui Technology Co., Yingtan 335000, China; weiwang@hbeu.edu.cn (W.W.); tj@xbrkj.com (J.T.); tjwen2025@163.com (K.T.); zhiqxu2024@163.com (Z.X.); 15273268006@163.com (F.Z.); jpdong0128@163.com (J.D.); yanxiachen2024@163.com (Y.C.)
[2] School of Chemistry and Materials Science, Hubei Engineering University, Xiaogan 432000, China
[3] Hubei Key Laboratory of Biological Resources and Environmental Biotechnology, Wuhan University, Wuhan 430000, China
* Correspondence: ceramic423@163.com (Z.F.); qincq@hbeu.edu.cn (C.Q.); Tel.: +086-0701-7091308 (Z.F.); +086-0712-2345464 (C.Q.)

Table of Contents

1. Figure S1. Effect of HEC on brightness and roughness of copper foils	2
2. Figure S2. Effect of HEC on tensile strength and elongation at break of copper foils	2
3. Figure S3. XRD analysis of copper foils prepared by adding different concentrations of HEC	3
4. Figure S4.The polarity map of copper foils prepared by adding 5.0 mg/L of HEC.	3
5. Figure S5. The NMR spectrum of sodium 3-((4,5-dihydrothiazol-2-yl)thio)propane-1-sulfonate (TPS)	4
6. Figure S6. The FTIR spectrum of sodium 3-((4,5-dihydrothiazol-2-yl)thio)propane-1-sulfonate (TPS)	5
7. Figure S7. The FTIR spectrum of 2-mercaptothiazoline	6

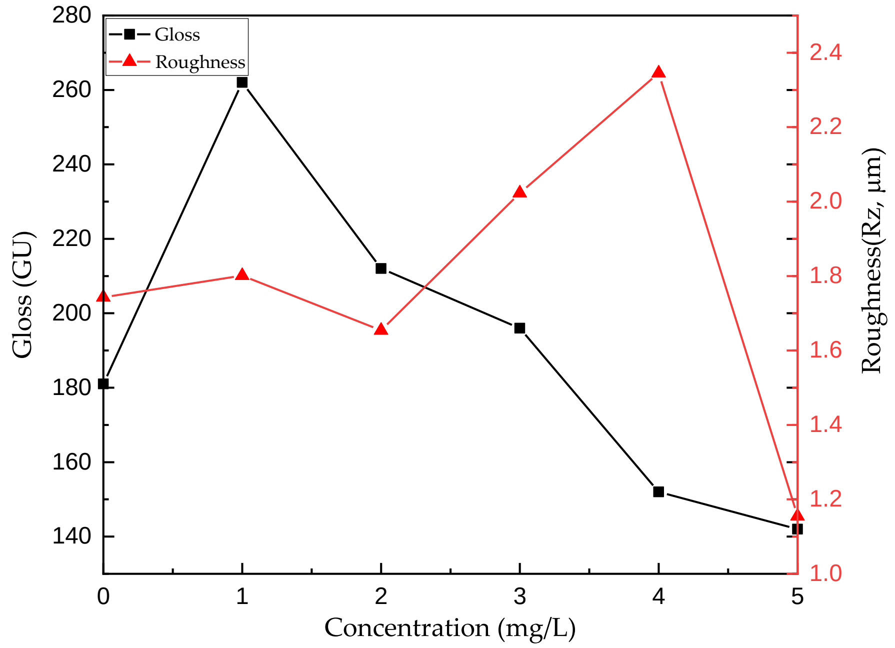

Figure S1. Effect of HEC on brightness and roughness of copper foils

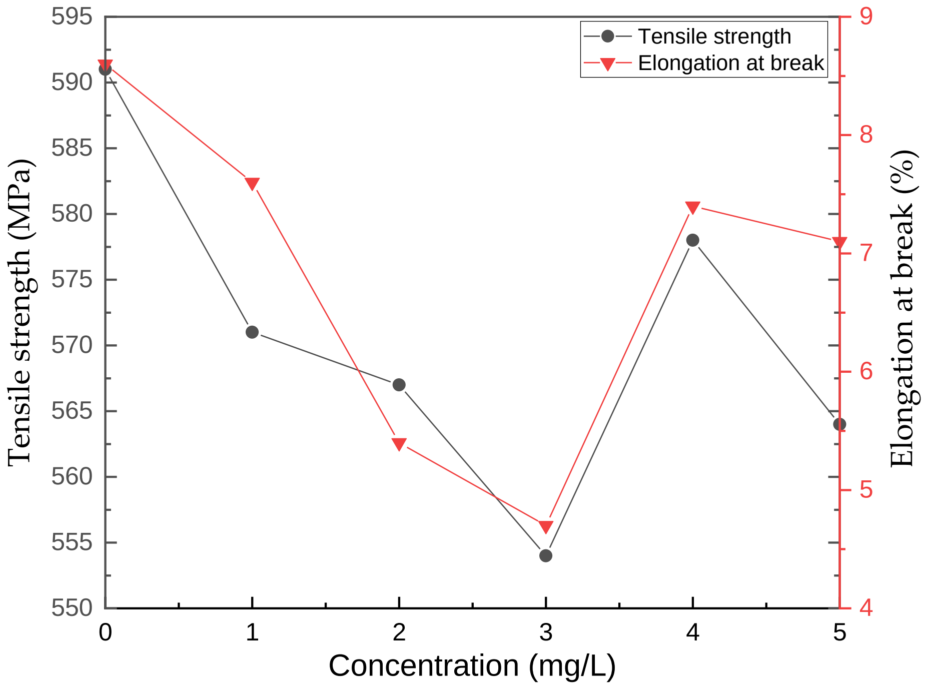

Figure S2. Effect of HEC on tensile strength and elongation at break of copper foils

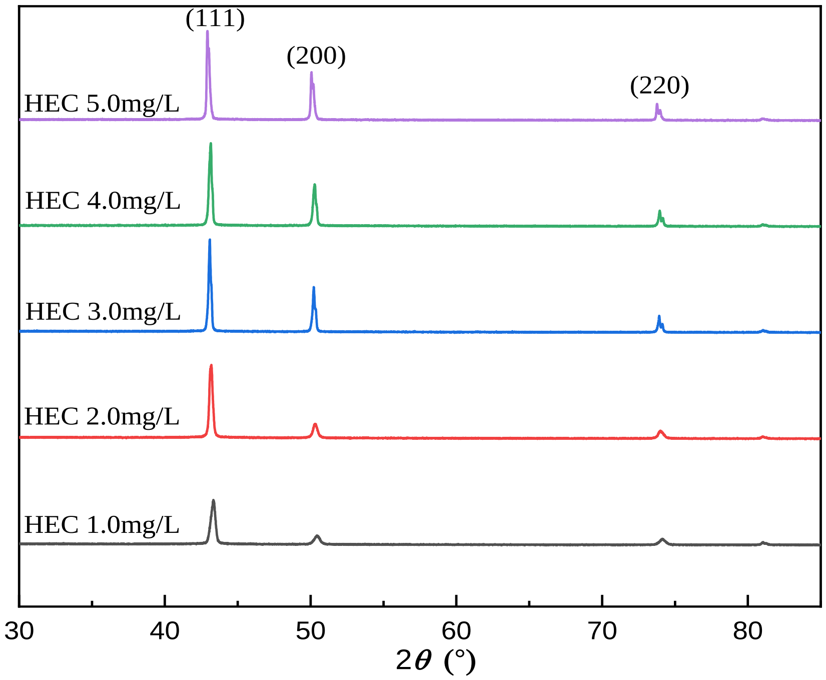

Figure S3. XRD analysis of copper foils prepared by adding different concentrations of HEC

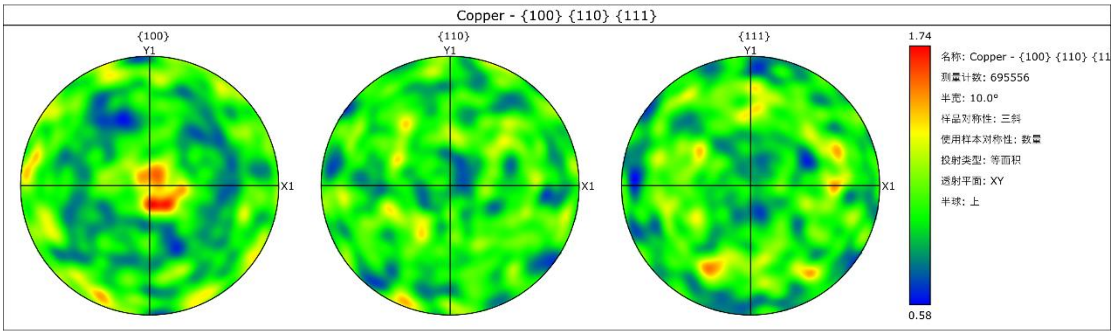

Figure S4.The polarity map of copper foils prepared by adding 5 mg/l of HEC.

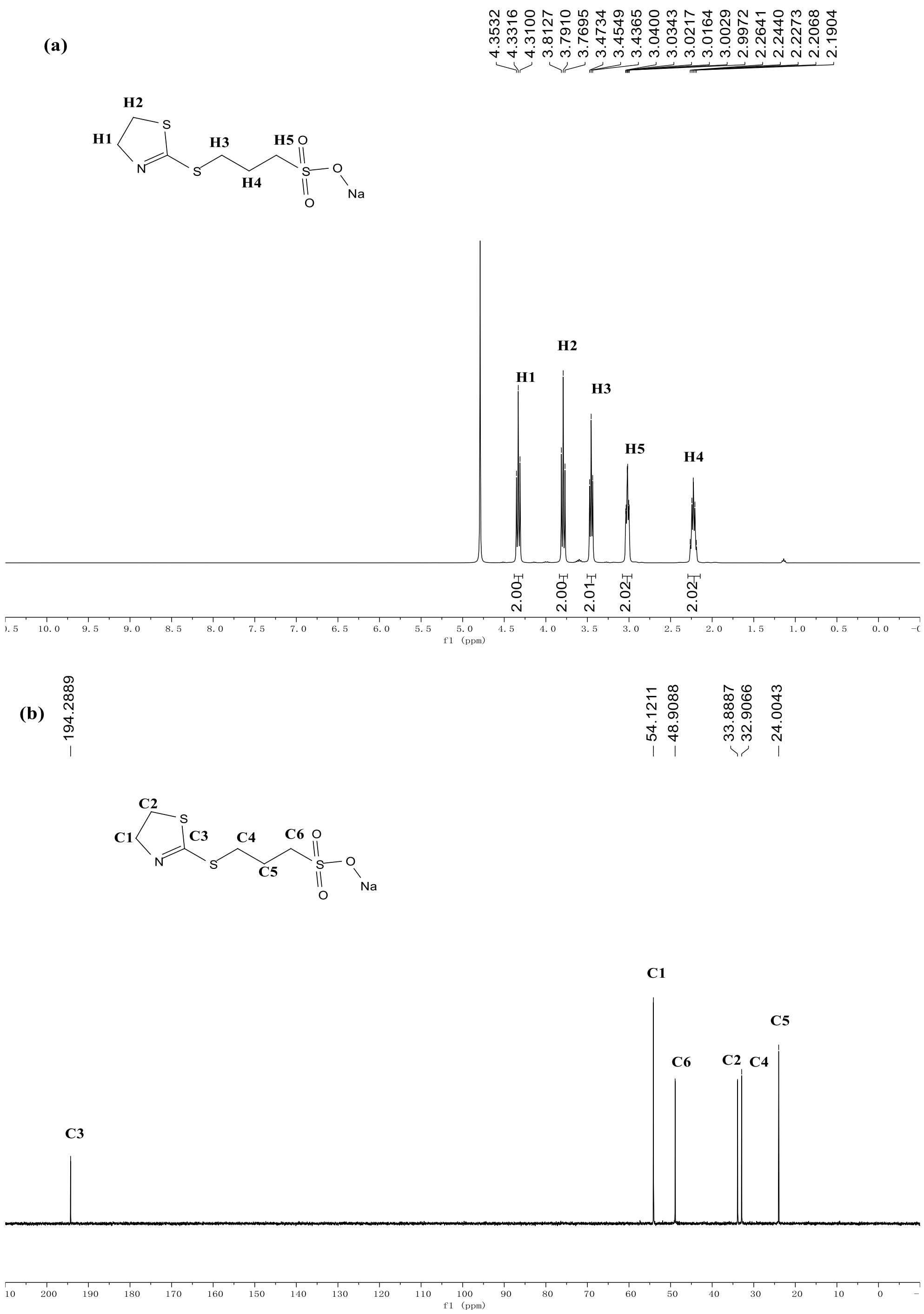

Figure S5 . The NMR spectrum of sodium 3-((4,5-dihydrothiazol-2-yl)thio)propane-1-sulfonate (TPS): (a) ^{1}H NMR and (b) ^{13}C NMR.

The IR spectra of TPS is shown in Figure S2 , and the IR spectra of 2-mercaptothiazoline is shown in Figure S1 in the supporting information. 3153 cm^{-1} and 2940 cm^{-1} should be the stretching vibration of the C-H group on the methylene group. 1638 cm^{-1} may be the absorption peak of the stretching vibration of C=N, 1219 cm^{-1} and 1156 cm^{-1} correspond to the asymmetric stretching peaks of the sulfonic acid group, 1018 cm^{-1} is the absorption band of the stretching vibration of C-S, and 940 cm^{-1} corresponds to the bending vibration peak of the five-membered heterocyclic ring. The results of IR spectroscopy also confirmed the successful synthesis of TPS.

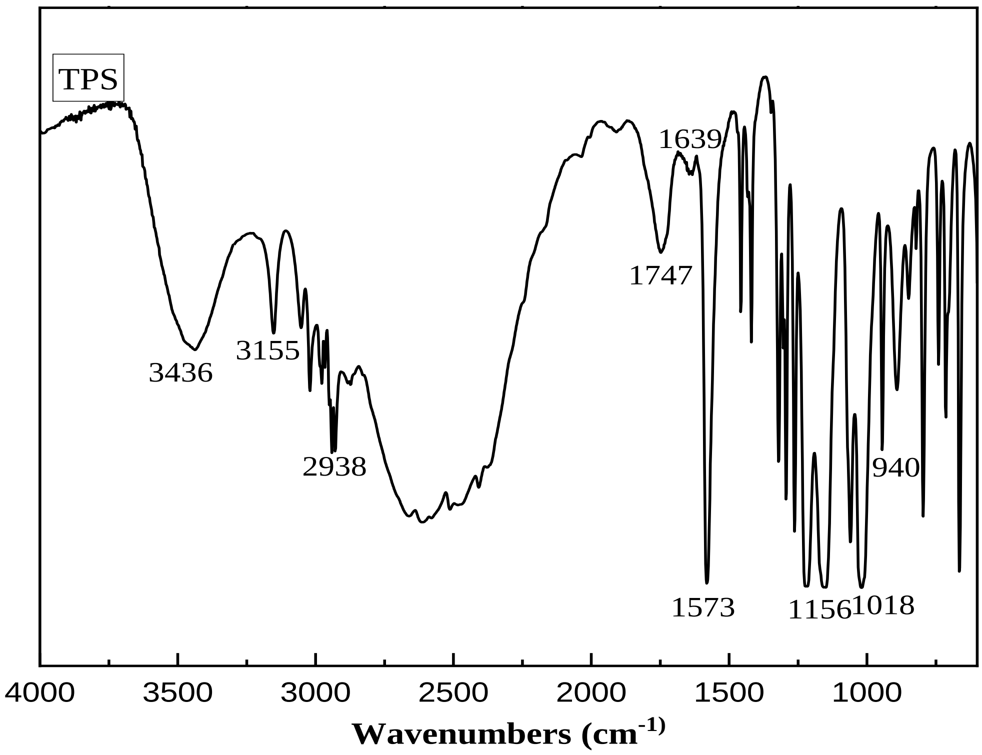

Figure S6. The FTIR spectrum of sodium 3-((4,5-dihydrothiazol-2-yl)thio)propane-1-sulfonate (TPS)

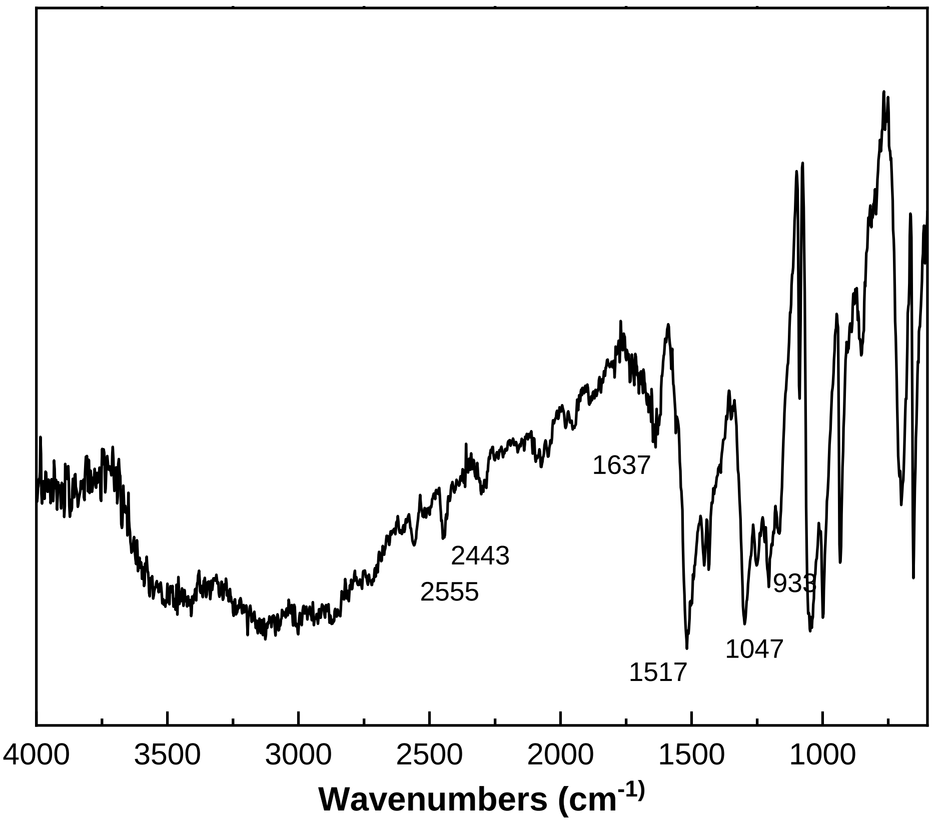

Figure S7. The FTIR spectrum of 2-mercaptothiazoline
